# Do the dynamics of vaccine programs improve the full immunization of children under the age of five in Cameroon?

**DOI:** 10.1186/s12913-020-05745-x

**Published:** 2020-10-15

**Authors:** Rodrigue Nda’chi Deffo, Benjamin Fomba Kamga

**Affiliations:** 1 Faculty of Economics and Management, University of Yaounde II, Yaounde, Cameroon; 2 Applied Microeconomics Research Laboratory, P.O. Box 14442, Yaounde, Cameroon; 3 IZA Institute of Labor Economics, Schaumburg-Lippe-Straße 5-9, 53113 Bonn, Germany

**Keywords:** Immunization, Childhood, Child health, Decomposition, Immunization coverage

## Abstract

**Background:**

Among the eight Millennium Development Goals (MDGs), three were devoted to health. Two amongst which MDG4 in relation to the reduction of infant mortality has not been achieved in Least Developed Countries (LDC). In Africa, a significant part of infant mortality is due to vaccine-preventable diseases administered free of charge by the Extended Program on Immunization (EPI). As such, in the “social equity” pillar of Sustainable Development Goals (SDG), the MDGs related to health have yet been taken into account. The achievement of these objectives requires an understanding of the immunization behavior of children under 5 years of age through an analysis of immunization dynamics between 1991 and 2011.

**Methods:**

We use data from Demographic and Health Surveys (DHS) of 1991, 1998, 2004 and 2011 carried out by the National Institute of Statistics (NIS). The module concerning EPI vaccines was administered to 3350, 2317, 8125 and 25,524 under 5 in 1991, 1998, 2004 and 2011 respectively. The Immunization analysis was made from the logistic model for complete immunization and the Oaxaca’s decomposition to assess the contribution of the unexplained part, which is that of the strategies/programs implemented between 1991 and 2011 by the EPI to improve immunization.

**Results:**

In general, children with vaccination card are more than 7 times likely to be fully immunized than their counterparts who do not have any. This result was higher in 1991 (approximately 57) and lowest in 2011 (5). In addition, the child’s birth order reduces his/her probability of being fully immunized and the impact increases with the latter’s birth order. On the other hand, the mother’s age as well as her level of education increase the child’s likelihood of receiving all basic vaccines. Moreover, the contributions of EPI partners in terms of immunization support as well as strategies to promote immunization through communication for development are of a particular importance in increasing immunization coverage. They significantly explain 67.62% of the 0.105 gain recorded within the 2011–2004 period and 72.46% of the 0.069 gain recorded within the 2004–1998 period.

**Conclusion:**

The contribution of EPI partner organizations is fundamental for the achievement of EPI objectives. Since they contribute to increase the likelihood of fully immunized children. The link with child immunization is done through the specific characteristics to the mother.

## Background

The United Nations Millennium Declaration in 2000 devoted 3 of the 8 goals^1^ adopted to health. This interest in health can be seen in the “social equity” pillar of the 2015 Sustainable Development Goals (SDGs), which recognizes health as a component of the population’s well-being and a factor of growth [1]. Based on these objectives, many organizations such as the World Bank, WHO, the United Nations International Childrens Emergency Fund (UNICEF), the Global Alliance for Vaccines and Immunization (GAVI) have mobilized to improve child health in terms of nutrition and immunization. Despite the deployment of these agencies in child health, there are still significant challenges to achieving universal coverage in developing countries [2]. Although the limited coverage of child immunization programs such as the EPI has reached 83% by 2014, child immunization remains a real problem in sub-Saharan African countries in general and in Cameroon in particular [3].

As a classic case of social dilemma, vaccination in view of its specificities can be analyzed as a gap between the willingness of public authorities to derive maximum benefit from it through the eradication of the disease and the perception of the general interest by the population [4]. From this point of view, many authors have devoted themselves to analyzing the demand for vaccination [4–7]. Unanimously, they find that the demand for vaccination is elastic to the prevalence of the disease. Hence, an individual will have less incentive to be vaccinated if the prevalence of the disease is low and the risk of getting the disease is lower than the cost of vaccination. These market failures tend to create forces that lead to inadequate demand, thus justifying government action through tax and subsidy mechanisms as an incentive. For example, basic vaccines for children under 5 years of age are administered free of charge as part of the EPI and campaigns are implemented to ensure that all children are vaccinated.

Despite the importance of incentives to achieve universal coverage, child immunization remains a real problem due to the high maternal illiteracy rate, weak health infrastructure, as well as its geographical distribution. Thus, empirical analyses on childhood immunization have focused more on demand factors to the detriment of immunization policies. These studies, mostly in low-income countries, focus on socio-economic, demographic and household characteristics to show the link between children’s immunization status and the mother’s education as well as her socio-economic status [8–13]. In terms of birth order, the trend observed is that early children are more likely to be fully vaccinated than their later-born siblings [9, 12]. Similarly, in some regions boys are more likely to be fully immunized than girls [8, 12].

Moreover, the difference in development strategies and in the definition of rural and urban areas creates ambiguity between studies regarding the effect of the residential environment [8, 9, 13]. Although the literature on the relationship between immunization and demographic, socioeconomic factors and household characteristics presents conclusive results, there are significant differences between countries and localities in the sources of inequalities in child immunization due to significant variations in structural, cultural and institutional settings [14]. The existence of these discriminatory factors justifies the implementation of specific immunization promotion strategies to stimulate maternal awareness about child health. However, the impact of these immunization promotion strategies implemented by countries and supported by public and private organizations to encourage populations to be immunized is not known.

In this article, we seek to contribute to the existing literature by examining the effect of support programs on the susceptibility of children to fully take basic vaccines in Cameroon. The objective of this paper is therefore twofold. Firstly we examine the determinants of complete immunization of children aged 12 to 59 months using logistic regression. More specifically, this paper evaluates the probability that a child will receive all the basic immunizations required by the WHO and administered within the framework of the EPI. It then decomposes the differences in probability of full immunization coverage between two study periods into a part attributable to differences in observed characteristics and an unexplained component that may reflect organizational differences in terms of the administrative and financial aspects of the body in charge of immunizing children.

We use data from the Demographic and Health Survey (DHS) carried out by the National Institute of Statistics (NIS) in 1991, 1998, 2004 and 2011. The use of data from these periods allows us to take into consideration the changes that the EPI has undergone on the administrative level and the adaptation of strategies to the difficulties encountered in achieving the objectives. In addition, the contribution of private and public organizations has not remained stable over time, but has fluctuated during the periods selected for the study. This assistance has undoubtedly affected the activities of the program, given that the EPI does not have its own fund for its operation. The analysis is structured around a presentation of EPI’s organization, the methodology used for the analysis, the results, the discussion and finally the conclusion.

### Organization of children’s immunization in Cameroon

The immunization system of children under 5 in Cameroon is organized in two parts: a paid and a free part. EPI manages the free part which concerns children aged 0 to 11 months. From the outset, it included six diseases^2^ recommended by WHO in 1984. But this program evolved with time given the introduction of the yellow fever vaccine in 2004, the viral hepatitis B vaccine in 2005, the *Haemophilus influenzae* type b vaccine in its form pentavalent DTP-HepB+Hib in February 2009, the pneumococcus vaccine in July 2011 and the rotavirus diarrhea vaccine in March 2014. These vaccines are administered according to the schedule presented in the following table (Table 1).

**Table 1:** Immunization Schedule for children less than 1 year

Diseases	Vaccines	Doses	Age
Tuberculosis	BCG	1	At Birth
Poliomyelitis	OPV	3	6, 10, 14 weeks
Diphtheria-Tetanus-Pertussis-Viral Hepatitis B-*Haemophilus influenzae* type b	DTP-HepB-Hib	3	6, 10, 14 weeks
Pneumococcal Disease	VPC13	3	6, 10, 14 weeks
Rotavirus diarrhea	Rotarix	1	6, 10 weeks
Measles	VAR	1	9 months
Yellow fever	VAA	1	9 months

Source: PEV [15]

EPI started in Cameroon in 1976 as a pilot project coordinated by the Organization for the Coordination of the fight against Endemic diseases in Central Africa (OCECA). With the Declaration of the Reorientation of Primary Health Care (DRPHC), the initial experimental program was integrated in the minimum activity package of all health facilities in the country and became operational in all regions of the country in 1982. The EPI has concentrated its activities around immunization incentive programs in order to achieve objectives pertaining to her membership to the Global Immunization Vision and Strategy (GIVS). Since the creation of a central body under the supervision of the Ministry of Public Health, the immunization of children is organized as a service delivery strategy and additional immunization accompanied by a communication system for behavior improvement.

Service delivery strategies are regulatory immunization sessions organized at the level of public or private health facilities according to a preset weekly program for 0–11 months children and pregnant women. They vary according to the proximity to health facilities. For populations located less than 5 km from the health facility or at most, an hour of walking, immunization at a permanent location is suggested i.e., in the health facility’s premises. However, for households located more than 5 km or beyond an hour of walking, the health facility organizes sessions outside their premises. This is done following an agreed schedule with the community concerned. Its frequency depends on the birth rate in the said area. If the locality is even further from the health facility, the mobile immunization strategy which consists of health professionals going to stay for several days in the area to perform immunizations and other health activities is used.

To compensate for non-immunized children due to remoteness and other reasons, supplementary immunization strategies refer to catch-up actions, immunization and response campaigns. With a sick child aged 0–59 months coming in for consultation, it is the responsibility of the health professional to check his/her immunization status and to complete it if necessary. This policy applies to all antigens except the BCG which is reserved solely for children less than a year. In addition to this, vaccination campaigns are organized for the eradication of poliomyelitis, the control of measles and the elimination of neonatal tetanus.^3^ The control of the yellow fever is done with the help of preventive response campaigns in the population of at least 9 months.

The success of these strategies is based on the communication system organized around three components which are advocacy, social mobilization and communication for behavior change. This technique is known under the slogan “*Communication for Development*” and represents the communication approach recommended for social mobilization activities.

Since 2006, advocacy and social mobilization have been oriented toward the quest for support by various authorities at all levels for immunization activities and the strengthening of immunization facilities. To this effect, team of three people have been set up in each region to ensure communication. Community radios are the main bodies with which the program has formal agreements. Collaboration with religious denominations, rural and public radio stations also contributed to mass immunization campaigns as well as routine immunizations.

Communication activities for behavior change consist in raising the awareness of families by the local mobilizers who go from door-to-door, disseminating messages about immunization by town criers via megaphones, using mobile amplified sound systems, making announcements and news releases in churches, mosques, schools, meeting places, broadcasting commercials and micro programs on rural and community radio stations. These activities are often held over the course of mass immunization campaigns during which health areas and districts have the means of contributing via communication focal points and local mobilizers, and also supporting rural and community radio stations.

Apart from health districts and areas that receive support for acceleration activities, the communication component for behavior change rarely benefits from specific funding because partners only fund specific items such as the purchase of vaccines and immunization equipment. As such, communication focal points and local mobilizers present in the vast majority of health districts and areas have only been active during supplementary immunization activities.

Despite the difficulties of the EPI regarding the administration of vaccines, the 2004–2010 period was is characterized by high levels of coverage. This performance can be explained beyond the initial strategies of the EPI by:

- The provision of GAVI Funds within the framework of the award per additional fully immunized child through the signing performance contracts with health districts from 2004;
- The implementation of the Reaching out to Every District (RED) approach which has been generalized in all health districts (HD) since 2005;
- Catching-up with non-immunized and under immunized children within the framework of Action Weeks for Child and Maternal Health and Nutrition (AWCMH);
- Catching-up in some HDs via the financial support of ENP + UNICEF funds and catalytic funds of WHO since 2008;
- The development of the Complete Multi Annual Plan (2007–2010);
- The Financial viability Plan from 2004. It facilitates the development of annual plans by highlighting the objectives set in accordance with GIVS-MDGS.

## Methods

### Data sources

The data used in this study were extracted from Demographic and Health Surveys (DHS) carried out by the National Institute of Statistics (NIS) in 1991, 1998, 2004 and 2011. The DHS are performed by the NIS in collaboration with its partners: the United Nations Fund for Population Activities (UNFPA), the United Nations International Children’s Emergency Fund (UNICEF), the World Bank and the United States Agency for International Development (USAID) as well as the technical assistance of ORC Macro. The investigation is stratified in such a way as to provide an adequate representation of urban and rural areas as well as the 12 study areas which correspond to the 10 administrative regions and cities of Yaoundé and Douala. The DHS provide information on fertility, family planning, maternal health, nutritional status of children, immunization status of children as well as infant and child mortality.

During the investigation, 3538 households were visited in 1991, 4697 in 1998, 10,462 in 2004 and 14,214 in 2011. In these households, 3871, 5501, 10,656 and 15,426 women aged 15–49 years were surveyed. These women enabled us to obtain information on 3350 children under the age of 5 in 1991, 2317 children under the age of 5 in 1998, 8125 children under the age of 5 in 2004 and 25,524 in 2011. In order to analyze full immunization, we restricted our sample of children to those aged at least 10 months i.e. who are deemed to have completed their compulsory immunizations. After processing the variables, we remain with a sample of 13,632 children, including 1677, 1119, 4278 and 6558 in 1991, 1998, 2004 and 2011 respectively.

### Determinants of child immunization status

The modeling is based on the assumption of heterogeneity amongst children in relation to their personal characteristics, the characteristics of the mother and that of the household. Individual preferences in terms of preventive health care are represented by a utility function because preventive actions are viewed as the consumption of goods that affect the risk of illness [16]. This utility function is estimated by a comparison between the benefit and cost perceived by parents, and depends on the characteristics of the child, the mother and the household [4, 5, 16]. Parents therefore choose a level of immunization to achieve the maximum utility possible. Given *U*_*ij*_ the said maximum utility for a given child *i* (*i* = 1, ..., *n*) when the parent chooses a prevention behavior *j*. This utility function may be broken down into a deterministic component *X*_*ik*_*β*_*jk*_ and a stochastic component *ε*_*ij*_ such that:

1 $$ {U}_{ij}={X}_{ik}{\beta}_{jk}+{\varepsilon}_{ij} $$

Where *X*_*ik*_ is a vector of *k* observable characteristics (bio-demographic characteristics of the mother and household characteristics) which constraints parents’ choice of the quality of their child [17], *β*_*jk*_ is a parameter vector when parents choose a level of prevention *j* and *ε*_*ij*_ is the error term. Consider a parent who must choose between full immunization (*FI*) and partial vaccination (*PV*) for his/her child, the utility gained from his/her choice is not observable. What is observable is the choice which is equal to 1 if *U*_*iFI*_ > *U*_*iPV*_ and 0 if *U*_*iFI*_ ≤ *U*_*iPV*_.

Thus, the probability that a parent chooses to fully immunize his/her child is given by:

2 $$ \Pr \left({y}_i=1|X\right)=\Pr \left({U}_{iFI}>{U}_{iPV}\right) $$

After handling and substituting *U*_*iFI*_ and *U*_*iPV*_ by their formula given in (1), this allows us to have:

3 $$ \Pr \left({y}_i=1|X\right)=\Pr \left({\mu}_i\le {X}_{ik}{\delta}_k|X\right)\kern0.5em X $$

Suppose that the error term *μ*_*i*_ follows a logistic distribution function *Λ*, the change in proportion when the predictor *X* increases by a unit designated by Odds ratio is given by the following relationship:

4 $$ \frac{\Pr \left({y}_i=1|X\right)}{\Pr \left({y}_i=0|X\right)}=\exp \left({X}_{ik}{\delta}_k\right) $$

The estimation of model (4) parameters is obtained by the maximum likelihood method. When the value of this quantity is higher than 1, the likelihood increases with the change. The estimate will be made for all periods put together and per period taken separately.

### The decomposition of full immunization

Here, the method is a technique commonly attributed to Blinder [18] and Oaxaca [19]. It is to decompose the intergroup differences in the average observations of a given variable based on differences due to endowments between these groups and that attributable to the effects of the said characteristics. According to this principle, the decomposition of the gap in a variable *U* between two periods can be formulated because of a regression and by using the *t*_2_ period as a reference as follows:

5 $$ {\overline{U}}^{t_2}-{\overline{U}}^{t_1}=\left({\overline{X}}^{t_2}-{\overline{X}}^{t_1}\right){\overset{\frown }{\beta}}^{t_2}+{\overline{X}}^{t_1}\left({\overset{\frown }{\beta}}^{t_2}-{\overset{\frown }{\beta}}^{t_1}\right) $$

Knowing that *U* is not observable, but rather a dichotomous variable equal to 1 if there is full immunization and 0 otherwise, Fairlie [20] uses an equivalent analysis to perform a decomposition in the presence of the estimates of a logit model. According to this approach, the decomposition in the case of non-linear models can be expressed in the following manner:

6 $$ {\overline{U}}^{t_2}-{\overline{U}}^{t_1}=\left[\sum \limits_{i=1}^{T_2}\frac{\varLambda \left({X}_i^{t_2}{\overset{\frown }{\beta}}^{t_2}\right)}{T_2}-\sum \limits_{i=1}^{T_1}\frac{\varLambda \left({X}_i^{t_1}{\overset{\frown }{\beta}}^{t_2}\right)}{T_1}\right]+\left[\sum \limits_{i=1}^{T_1}\frac{\varLambda \left({X}_i^{t_1}{\overset{\frown }{\beta}}^{t_2}\right)}{T_1}-\sum \limits_{i=1}^{T_1}\frac{\varLambda \left({X}_i^{t_1}{\overset{\frown }{\beta}}^{t_1}\right)}{T_1}\right] $$

With ${\overline{U}}^t$ the average probability to declare oneself fully immunized in the population of the period *t* (*t* = *t*_1_, *t*_2_) and *Λ* the distribution function of logistic distribution. The decomposition associated with Eq. (6) uses the second period as a reference since estimated coefficients on the population of this period are used to weight the first term of the expression meanwhile the distribution of the population’s characteristics of the first period is used to weight the second term. The choice of the population of the second period as a reference population thus suggests changes in immunization behavior to the detriment of the population of the first period [18, 19]. It suggests that discrimination is made in favor of the second period’s population.

The method proposed by Fairlie allows the assessment of the relative contribution of each variable to the difference in the average probability of being fully immunized. This relative contribution of an observable characteristic *X*_1_ given by Eq. (7) can be positive or negative. When it is negative, this suggests that the variable concerned contributes to the reduction of the full immunization difference which is attributed to a difference in the distribution of observable characteristics between the populations of the two periods.

7 $$ \frac{1}{T_1}=\sum \limits_{i=1}^{T_1}\varLambda \left({\hat{\beta}}_o^{t_2}+{X}_{1i}^{t_2}{\hat{\beta}}_1^{t_2}+\cdots +{X}_{ki}^{t_2}{\hat{\beta}}_k^{t_2}\right)-\varLambda \left({\hat{\beta}}_o^{t_2}+{X}_{1i}^{t_1}{\hat{\beta}}_1^{t_2}+\cdots +{X}_{ki}^{t_2}{\hat{\beta}}_k^{t_2}\right) $$

Thus, the sum of the relative contributions of each characteristic represents the total immunization difference attributed to differences in the distribution of observable characteristics between the two populations. The immunization difference not explained by the distribution of observable characteristics represents the share of the total immunization difference due to differences in the estimated coefficients. This second difference shows that with identical observable characteristics, the population of the second period does not reflect the same immunization status as the population of the first period because of a different effect of observable characteristics [20] or the dynamics of the EPI.

### Variables of the study and a few descriptive statistics

A child is born with passive immunity from the mother and his/her consumption of breast milk; but that loses its effectiveness as the child grows. This loss of effectiveness of passive immunity must be compensated for by immunization which provides the child with active immunity. As such, when a vaccine’s due date arrives, implying that the child can already contract the disease prevented by this vaccine, not taking the vaccine is a health risk for the child [21]. Thus, some studies have analyzed the immunization of children in terms of antigen-intake [22]. On the other hand, given the exclusive character of vaccines, others think it is important that the child is fully vaccinated [13, 23]. In accordance with WHO recommendations, children are considered fully immunized if they have received the basic EPI vaccines: the BCG vaccine against tuberculosis, three doses of DTP^4^ against diphtheria, tetanus and pertussis, three doses of the polio vaccine and measles vaccines. Our study will analyze full immunization by focusing on basic vaccines (BCG, polio, DTP and VAR) in order to allow for comparison between the four series of surveys.

According to Patra [23] and Asuman, Ackah [13], independent variables are grouped into: bio-demographic characteristics (vaccination card, gender, birth order and the birth weight, age of the child); the mother’s characteristics (mother’s age, religion, level of education, marital status and exposure to the media, the mother’s occupation and the level of prenatal visits); and the household’s characteristics (the area of residence and the size of the household). The specification of variables is done in Table 2.

**Table 2:** Specification and descriptive statistics of the variables of the study

Variables	Specifications	Full immunization (column %)			
		1991	1998	2004	2011
**Bio-demographic characteristics**					
**Vaccination card**					
Card	1 if the child has a vaccination card: 0 otherwise	99.35	98.38	93.99	93.61
No card	1 if the child does not have a vaccination card: 0 otherwise	0.65	1.62	6.01	6.39
**Gender of the child**					
Male	1 if the child is male: 0 otherwise	53.00	51.16	49.54	48.74
Female	1 if the child is female; 0 otherwise	47.00	48.84	50.46	51.26
**Child’s birth order**					
Order 1	1 if the child is of Order 1; 0 otherwise	14.75	22.45	21.56	19.96
Order 2	1 if the child is of Order 2; 0 otherwise	17.02	18.06	19.40	20.15
Order 3	1 if the child is of Order 3; 0 otherwise	15.40	13.66	16.02	17.30
Order 4	1 if the child is of Order 4; 0 otherwise	13.94	13.43	12.53	13.35
Order 5	1 if the child is of Order 5; 0 otherwise	10.53	12.04	9.50	10.04
Order 6	1 if the child is of Order 6; 0 otherwise	9.40	7.41	7.03	7.07
Order 7 and beyond	1 if the child is of Order 7; 0 otherwise	18.96	12.96	13.96	12.13
**Birth weight**					
Low	1 if the child is born with less than 2500 g; 0 otherwise	8.43	7.87	8.01	8.35
high	1 if the child is born with more than 2500 g; 0 otherwise	67.26	66.67	60.57	66.20
Not weighed	1 if the child was not weighed at birth: 0 otherwise	12.80	22.45	26.13	22.63
Does not know	1 if the mother does not know the child’s weight: 0 otherwise	11.51	3.01	5.29	2.83
**Mother’s characteristics**					
**Mother’s age**					
15–19	1 If the mother is in the 15–19 age group: 0 otherwise	3.89	5.56	4.93	4.03
20–24	1 If the mother is of the 20–24 age group: 0 otherwise	18.48	26.62	24.38	20.78
25–29	1 If the mother is of the 25–29 age group: 0 otherwise	29.66	29.17	27.98	29.34
30–34	1 If the mother is in the 30–34 age group: 0 otherwise	23.18	21.06	20.79	22.76
V35–39	1 If the mother is in the 35–39 age group: 0 otherwise	15.88	12.27	12.47	14.90
40–49	1 If the mother is in the 40–49 age group: 0 otherwise	8.91	5.32	9.45	8.19
**Religion**					
Traditional Christian	1 if the religion is catholic or protestant: 0 otherwise	84.28	82.18	75.10	78.84
Reformist religion	1 if the mother is a Muslim or another: 0 otherwise	15.72	9.26	13.96	15.50
No religion	1 if the mother has no religion: 0 otherwise	0	8.56	10.93	5.66
**Level of education**					
No level	1If the mother has no level of education: 0 otherwise	19.94	16.20	16.02	13.76
Primary	1If the mother has a primary level of education: 0 otherwise	45.22	45.14	50.41	46.86
Secondary	1If the mother has a secondary level of education: 0 otherwise	32.58	37.73	31.78	35.57
Higher education	1If the mother has a higher education level: 0 otherwise	2.27	0.93	1.69	3.81
**Marital status**					
Not in a relationship	1 if the mother is single: 0 otherwise	10.05	17.13	9.80	13.82
In a relationship	1 if the mother is in a relationship: 0 otherwise	89.95	82.87	90.20	86.18
**Media exposure**					
Not exposed	1 if the mother has no media exposure: 0 otherwise	31.77	49.77	32.75	25.67
Limited exposure	1 if the mother has limited media exposure: 0 otherwise	25.12	24.07	26.75	25.78
High exposure	1 if the mother is highly exposed to the media: 0 otherwise	43.11	26.16	40.50	48.55
**Mother’s occupation**					
For the family	1 if the mother works for the family: 0 otherwise	8.59	2.78	11.14	6.47
For others	1 if the mother works for others: 0 otherwise	17.34	7.41	7.80	10.47
Self-employed	1 if the mother is self-employed: 0 otherwise	74.07	89.81	81.06	83.06
**Prenatal visits**					
No follow-up	1If the mother had no prenatal follow-up: 0 otherwise	8.27	10.19	4.72	2.91
Incomplete follow-up	1If the mother had less than 4 follow-ups during the pregnancy: 0 otherwise	20.58	20.14	11.60	11.94
Complete follow-up	1If the mother had at least 4 follow-ups: 0 otherwise	71.15	69.68	83.68	85.15
**Characteristics of the household**					
**Place of residence**					
Urban	1 if the household resides in an urban area: 0 otherwise	62.24	40.28	38.66	41.56
Rural	1 if the household resides in an urban area: 0 otherwise	37.76	59.72	61.34	58.44
**Household size**^**a**^	Taille du ménage en continue	9.69 (5.85)	8.41 (4.03)	7.76 (4.44)	7.58 (4.09)
**Number of observations**		1677	1119	4278	6558

Source: from Demographic and Health Surveys of 1991, 1998, 2004 and 2011. The variables in parentheses are standard deviations

^a^Family size in continuous

Factors not observed both at the household and community levels not only affect fertility choices, but immunization as well and makes the household size endogenous. This endogeneity of household size is corrected using “non-self-mean clusters”. It involves the replacement of individual observations by averages at the community level (clusters). This method involves assigning an individual *i* from a community with size *N* the average of *N* − *i* other members of the community, which allows the complete elimination of the possibility of that individual to be affected by his/her personal preferences. To cancel scale effects, the *household size* variable will be composed using logarithm.

## Results

### Descriptive statistics of the study variables

Table 2 presents the descriptive statistics of the variables used in this study. These statistics which describe the situation of fully immunized children are grouped by period and according to specificity. Concerning the group of bio-demographic characteristics, the vaccination card which proves to be a fundamental reminder of the vaccination schedule and status of the child is very present for fully immunized children. The percentage of fully immunized children who possess a vaccination card has decreased by approximately 1 point between 1991 and 1998 from 99.35 to 98.38% and by about 5 points in 2004 (93.99%) and 2011 (93.61%). Regarding the child’s gender, the trend varies from one period to another. In 1991 and 1998, the proportion of fully immunized males was superior to that of females and the reverse is observed in 2004 and 2011. Children at the second position among siblings are the most represented among fully immunized children in 1991 and 2011. Meanwhile in 1998 and 2004, it is rather those occupying the first position. A downward trend is observed in the proportion of fully immunized children in relation to the birth order of the latter. In our sample, children born in low birth weight are the least represented. Therefore, they are also the least fully immunized with a proportion close enough between the periods. On the other hand, those born in high weight are fully immunized by more than 60% with a peak of 67.26% in 1991.

The mother is most often responsible for the child. An overview of her characteristics shows that those that fall in the 25–29 years age group are the most represented among fully immunized children. This proportion is 29.66% in 1991, 29.17% in 1998, 29.34% in 2011 and lowest in 2004 (27.98%). In contrast, women in the 15–19 years age group are the least represented with a proportion varying between 3.89% in 1991 and 5.66% in 1998. With regard to the mother’s religion, mothers who fully immunize their children are Catholic or Protestant and this by 84.28% in 1991, 82.18% in 1998, 75.10% in 2004 and 78.84% in 2011. Meanwhile those with no religion are the least represented. In addition, all mothers in 1991 have a religion. For the level of education, mothers with a higher level of education are the least represented among fully immunized children. The proportion is 3.81% in 2011 and less than 1% in 1998. Meanwhile, those who fully immunize their children generally have a primary level of education followed by those with a secondary level education. In addition, it is observed that approximately 20% of mothers who fully immunize their children are not educated. Generally, mothers are in a relationship by 89.95% in 1991, 82.87% in 1998, 90.20% in 2004 and 86.18% in 2011. Regarding the level of media exposure, it varies from one period to another; in 1991, 2004 and 2001, the majority of women having fully immunized their children are very exposed by 43.11, 40.50 and 48.55% respectively. They are mostly self-reliant with more than 74% in self-employment and have completely followed-up with prenatal visits for at least 69% of them.

For household characteristics, the distribution of households according to place of residence shows that fully immunized children were more numerous in urban areas in 1991 with a proportion of 62.24%. On the other hand, this trend was reversed from 1998 with 40.28% located in urban areas, 36.66% in 2004 and 41.56% in 2011. The household size decreases from 1991 to 2011, from an average of 10 persons to 8 persons.

### Determinants of the full immunization of children

Table 3 presents the Odds ratio of logistic regressions of full immunization behavior. The results show a significant difference in the probability of the full immunization of children between the different periods. In a specific way, compared to children surveyed in 1991, those of 2004 and 2011 are by 12 and 60% respectively, more likely to be fully immunized. In addition, the analysis by period shows that the effect of variables differs from one period to another and could be a source of the difference in behavior. Also, the evolution of the health system as well as health promotion programs that Cameroon has known since the beginning of the1990s could have changed the population’s behavior of recourse to care to the point of making them more aware of the importance of immunization. This difference in the immunization behavior arising from the difference in the effect of observable characteristics will be presented for each group of variables: the bio-demographic characteristics of the child, the characteristics of the mother and those of the household. The models are significant at the 1% threshold when the analysis is made for all periods as well as specifically per period.

**Table 3:** Determinants of full immunization

Variables	Odds Ratio				
	General	1991	1998	2004	2011
**Bio-demographic characteristics**					
**Vaccination card**					
No card *(ref)*					
Card	7.576 (0.473)***	56.914 (29.075)***	32.383 (12.912)***	8.738 (0.967)***	5.639 (0.467)***
**Gender of the child**					
Male *(ref)*					
Female	1.023 (0.039)	1.056 (0.131)	0.867 (0.123)	1.057 (0.072)	1.016 (0.056)
**Child’s birth order**					
Order 1 *(ref)*					
Order 2	0.961 (0.064)	1.016 (0.243)	0.761 (0.180)	0.869 (0.101)	1.009 (0.095)
Order 3	0.844 (0,063) **	0.844 (0.224)	0.670 (0.183)	0.761 (0,100) **	0.889 (0.093)
Order 4	0.739 (0.061)***	0.695 (0.197)	0.850 (0.264)	0.670 (0.099)***	0.725 (0.085)***
Order 5	0.653 (0.060)***	0.637 (0.198)	0.776 (0.255)	0.621 (0.102)***	0.627 (0.082)***
Order 6	0.608 (0.063)***	0.651 (0.213)	0.667 (0.262)	0.566 (0.106)***	0.609 (0.089)***
Order 7 and beyond	0.557 (0.056)***	0.614 (0.202)	0.534 (0,202) *	0.406 (0.074)***	0.639 (0.093)***
**Birth weight**					
Other *(ref)*					
Low	1.041 (0.078)	1.326 (0.330)	1.457 (0.419)	0.956 (0.125)	1.020 (0.108)
**Mother’s characteristics**					
**Mother’s age**					
15–19 *(ref)*					
20–24	1.435 (0.132)***	1.198 (0.377)	2.245 (0.664)***	1.169 (0.192)	1.575 (0.209)***
25–29	1.970 (0.196)***	1.961 (0.682) *	2.502 (0.835)***	1.647 (0.294)***	2.131 (0.301)***
30–34	2.458 (0.269)***	1.507 (0.560)	3.634 (1.391)***	1.874 (0.368)***	3.123 (0.487)***
35–39	2.809 (0.340)***	2.703 (1.112) **	3.624 (1.577)***	2.077 (0.457)***	3.392 (0.577)***
40–49	2.936 (0.389)***	2.703 (1.192) **	3.225 (1.596) **	3.469 (0.835)***	2.835 (0.528)***
**Religion**					
Traditional Christian *(ref)*					
Reformist religion	0.716 (0.038)***	0.900 (0.154)	0.577 (0.130) **	0.794 (0.077) **	0.687 (0.050)***
No religion	1.160 (0.091) *	–	1.665 (0.483) *	1.346 (0.163) **	0.838 (0.095)
**Level of education**					
No level *(ref)*					
Primary	1.347 (0.076)***	2.082 (0.363)***	1.208 (0.266)	1.432 (0.144)***	1.246 (0.102)***
Secondary	1.426 (0.098)***	2.413 (0.548)***	1.571 (0.404) *	1.647 (0.205)***	1.251 (0.123) **
Higher education	1.905 (0.336)***	4.126 (3.373) *	1.693 (1.567)	2.858 (1.102)***	1.649 (0.357) **
**Marital status**					
In a relationship *(ref)*					
Not in a relationship	0.915 (0.055)	0.627 (0.126) **	0.935 (0.184)	0.741 (0.083)***	1.119 (0.096)
**Media exposure**					
Not exposed *(ref)*					
Limited exposure	1.006 (0.052)	0.914 (0.148)	0.907 (0.162)	0.779 (0.070)***	1.214 (0.092) **
High exposure	1.156 (0.063)***	1.405 (0.259) *	1.407 (0.299)	0.823 (0.081) **	1.334 (0.102)***
**Mother’s occupation**					
Self-employed *(ref)*					
For the family	1.007 (0.069)	1.036 (0.211)	0.524 (0.203) *	1.190 (0.133)	0.970 (0.103)
For others	0.938 (0.072)	1.086 (0.264)	0.956 (0.307)	0.913 (0.130)	0.848 (0.089)
**Prenatal visits**					
No follow-up *(ref)*					
Incomplete follow-up	1.362 (0.121)***	1.335 (0.292)	0.983 (0.268)	1.110 (0.182)	1.569 (0.225)***
Complete follow-up	2.020 (0.159)***	1.745 (0.351)***	1.215 (0.304)	1.712 (0.243)***	2.363 (0.304)***
**Characteristics of the household**					
**Place of residence**					
Urban *(ref)*					
Rural	0.936 (0.042)	0.491 (0.068)***	0.873 (0.142)	1.026 (0.081)	1.027 (0.067)
**Household size**	0.589 (0.058)***	0.723 (0.223)	1.150 (0.461)	0.711 (0.128) *	0.479 (0.065)***
**Period**					
1991 *(ref)*					
1998	0.935 (0.083)				
2004	1.122 (0.077) *				
2011	1.601 (0.105)***				
**Constant**	0.123 (0.031)***	0.013 (0.012)***	0.010 (0.010)***	0.133 (0.058)***	0.273 (0.092)***
**Log likelihood**	− 7895.862	− 780.704	− 595.435	− 2512.031	− 3878.620
**Number of observations**	13.632	1.677	1.119	4.278	6.558
**LR chi2**	3100.32 ***	644.98 ***	301.77 ***	872.35 ***	1237.22 ***
**Pseudo** ***R***^**2**^	0.164	0.292	0.202	0.148	0.138

Source: From Demographic and Health Surveys of 1991, 1998, 2004 et 2011

The variables in parentheses are standard deviations *** (**) [*] represent the significance thresholds at 1% (5%) [10%] respectively

With respect to bio-demographic characteristics, between 1991 and 2011, the possession of the child’s vaccination card which shows the immunization schedule and the date of the next appointment increases by more than 7 times the chances of the child to be fully immunized This effect displays some disparities depending on the periods and approaches 57% for children surveyed in 1991. However, there is no gender-based disparity in the immunization status of children in Cameroon. This last result is consistent with that of Asuman, Ackah [13] concerning Ghana. On the other hand, evidence of gender-based differences in immunization status among children have been widely reported by studies of South Asian countries where there is a strong preference for male children [24, 25].

The results show that the probability for the child to be fully immunized decreases with an increase in birth order. As a whole, this effect is significant for the 2004 and 2011 periods. This result suggests a certain weariness of mothers as births increase. On the other hand, Antai [26] who obtained the same results in Nigeria postulates that this reflects competition for parental care between brothers and sisters. In addition, this result validates the theory of the dilution of resources suggested by Blake [27]. According to this theory, as the number of children increases, the resources allocated to each decrease such that children of higher birth order find themselves with less resources than their elders at that age.

The significant relationship between maternal characteristics and the probability of the child’s full immunization indicate the importance of those characteristics for the child’s immunization status. Just as Asuman, Ackah [13] and Adedokun, Uthman [28], there is a positive relationship between the mother’s age and the likelihood that the child is fully immunized. The impact increases as the woman increases in age; this is true for the whole population as well as for each period. Given the ambient poverty in Cameroon, these results may reflect the fact that the young moms are generally from poor families and are forced to work to take care of their offspring alone due to the abandonment of fathers. In 2011, the percentage of mothers who were raising their children alone was estimated at 19.53% and this was highest in the 15–19 age group (32.46%) [29]. The effects of the mother’s age therefore highlight the community’s attitude vis-a-vis the latter. This state of things is confirmed by the effect of marital status. Compared to women in a relationship, not being in a relationship generally reduces the likelihood of full immunization, though this is not always significant.

With respect to the mother’s religion, the results remain mixed according to study period. They suggest the presence of significant religious effects on the likelihood of full immunization as a whole, in 1998, 2004 and 2011. As a whole, full immunization has decreased by 29%, with 43% in 1998, 21% in 2004 and 32% in 2011 for children whose mothers belong to reformist religions including Islam. Meanwhile, not belonging to any religion has rather increased the likelihood of full immunization. This effect of reformist religions that is contrary to the results of Asuman, Ackah [13] in Ghana, testifies of 2 things for the case of Cameroon. Firstly, reformist religions in Cameroon prefer miracles through prayers. Secondly, the north of Cameroon is par excellence the area where Islam prevails and is also the area where the health system is poorly developed with a very low concentration of health facilities.

Many empirical studies have shown that the improvement of maternal education allows an improvement of the child’s health and immunization [30, 31]. These considerations are at the core of the promotion of women’s education worldwide and particularly in developing countries. The results of our study move in this direction by showing that the likelihood of full the immunization of children increased by 35% when women had a primary level of education compared to those with no education and this goes up to 90% for those who have a higher level of education. We also notice that the impact of education is higher in 1991 compared to other periods. This is because, compared to children whose mothers have no education, those whose mothers have a primary level of education are twice more likely to be fully immunized and 4 times for those with a higher level of education.

Raising the awareness of populations to health and immunization usually goes through communication channels such as the radio, television, newspapers. This is the reason why being exposed to these means of communication could be advantageous to the susceptibility of the child to be fully immunized. In addition, results in relation to media exposure are ambiguous. Meanwhile, as a whole, the likelihood of children whose mothers are very exposed to the media to be fully immunized has increased by 15%, in 2004 we rather observe a decrease of 18%. The plausible explanation to this ambiguity would be the actual use of these media to raise the awareness of populations. In Cameroon, there is almost no program devoted to the promotion of health on the different channels covering the country.

Antenatal visits during pregnancy increase the likelihood of children to be born in good health as well as their likelihood to be immunized during their first years of life [32]. National and international institutions recommend that women follow at least 4 antenatal visits during the 9 months of pregnancy. These different visits serve as an opportunity for the woman to follow-up with the evolution of her pregnancy, take the vaccines required, but also to be informed on the immunization of the child when he/she will be born. This is the reason why in Cameroon, in addition to a few security and behavior tips, antenatal check-up records have the immunization schedule of the child. As a whole and in 2011, the importance of prenatal visits here is seen through the fact that children whose mothers have followed at least the 4 recommended prenatal visits are 2 times more likely to be fully immunized. This result reflects the effects of the improvement of access to and use of health services on infant health. The correction of the gap in terms of access to health in rural areas through mobile immunization campaigns is such that rural areas do not have a significant effect on the likelihood of full immunization of children after 1991. In addition, the likelihood of full immunization has decreased from 41% as a whole for each additional person and by 51% in 2011.

### Decomposition of inequalities in the immunization of children by period

A summary of the various sources of inequalities between the periods of immunization coverage of children in Cameroon obtained from the decomposition analysis is presented in Table 4. We analyze the inequalities for full immunization between the 1998–1991, 2004–1998 and 2011–2004^5^ periods with reference periods being the years 1998, 2004 and 2011 respectively. The consideration of these years as reference assumes that discrimination between the periods chosen is made in favor of the reference. The results reveal the existence of disparities between periods as concerns the likelihood that a child completely takes basic vaccines.

**Table 4:** Summary results of Oaxaca Blinder’s decomposition

Source:	1998–1991	2004–1998	2011–2004
Period 1	0.386 (0.018)***	0.455 (0.008)***	0.561 (0.006)***
Period 2	0.368 (0.013)***	0.386 (0.016)***	0.455 (0.008)***
Difference	0.018 (0.021)	0.069 (0.018)***	0.105 (0.010)***
Endowments/explained	0.012 (0.018)	0.015 (0.015)	0.029 (0.005)***
Coefficients/unexplained	−0.002 (0.019)	0.050 (0.016)***	0.071 (0.009)***
Cofficients/unexplained (%)	11.25	72.46	67.62
Observations	2796	5397	10,836

Source: from Demographic and Health Surveys of 1991, 1998, 2004 et 2011

The variables in parentheses are standard deviations *** (**) [*] represent the significance thresholds at 1% (5%) [10%] respectively

The average probability that a child receives all basic vaccines in 1998 was 0.386, against 0.368 in 1991. The difference in the probability of full immunization between the two periods is not significant. This statistical equality could be due to the fact that all measures implemented at the beginning of the 90s in terms of primary health care promotion policies did not have the expected effect [33]. In addition to that, the devaluation of the FCFA in 1994 which contributed in impoverishing the population to the point of making health care a priority of second order for the latter.

On the other hand, the difference in the probability of full immunization of children between 1998 and 2004 is 0.069 and the latter is significant at 1%. Concerning the source of the disparity, the results indicate that the endowment or the effect explained contributes to approximately 27% to the gap, but is not significant. In fact, the difference in the characteristics of children between 1998 and 2004 does not favor any of the two groups. However, the coefficient or the unexplained effect favors the children of 2004. This result implies that programs and immunization strategies put in place between 1998 and 2004 improve the immunization of children in 2004 for the same level of characteristics as those of 1998. This is due to several phenomena at the institutional level as well as the community level. In order to be reassured of funding to increase the implementation of its activities, an Inter-Agency Coordination Committee (IACC) chaired by the Minister of Public Health was put in place in 1998 with the role of coordinating and searching for funding. In addition, the EPI was placed under the responsibility of a Central Technical Group attached to the Cabinet of the Minister of Public Health in 2002 to give him a good visibility in order to have a positive impact on the achievement of its objectives. At the community level, the liberalization of the press at the beginning of the 1990s saw a rise toward the end of the said decade of many community radio stations which are close to the population and enabling the conveyance of information on vaccination campaigns.

In 2011 the average probability of full immunization of children increased to 0.561, higher by 0.105 to that of 2004. The origin of this disparity is assigned to approximately 32% to the endowments of the children. In fact, the differences in characteristics favor the children of 2011. This suggests that on average, the children of 2011 have endowment levels higher than their counterparts in 2004. The unexplained effect (0.071) which represents 67.62% of the total difference also favors children of 2011 and is higher by 2 points to that of the 2004–1998 (0.050) period. This result implies that the additional inputs in terms of accompaniment from which the EPI benefited between these two periods have been a plus for the immunization of children. Beyond the initial strategies of the EPI, from 2004, the program has benefited from:

- The provision of GAVI funds within the framework of the award per additional fully immunized child through the signing performance contracts with health districts from 2004;
- The implementation of the Attaining Every District approach which was generalized in all health districts since 2005;
- Catching-up with non-immunized and under-immunized children within the framework of SASNIM;
- Catching-up in some health districts by the financial support of EPI + UNICEF funds and catalytic funding of WHO from 2008.

The contribution of each group of characteristics to the gap between periods in the full immunization of children is presented in Table 5. The decomposition shows that the characteristics of the Child and the household size are the main factors that contribute to the widening of the gap between the 2011–2004 period regarding the likelihood for a child to be fully immunized. On the other hand, the mother’s characteristics contribute to the unexplained component or discrimination in favor of the children of the 2011 period. This means that in 2011, the mother’s conditions were more appropriate for the immunization of children than in 2004. In addition, the negative and significant sign of children’s characteristics rather reduced the effect of implemented programs in the 2004–1998 period.

**Table 5:** Source of the contribution of inequalities in full immunization between periods

Characteristics	1998–1991		2004–1998		2011–2004	
	Endowment	Return	Endowment	Return	Endowment	Return
Child	0.009 (0.012)	0.006 (0.063)	−0.011 (0.028)	−0.103 (0.032)***	0.026 (0.004)***	−0.039 (0.031)
Mother	0.006 (0.008)	0.005 (0.056)	0.026 (0.017)	−0.026 (0.044)	0.003 (0.003)	0.129 (0,054) **
Rural	−0.005 (0.004)	−0.003 (0.038)	0.001 (0.002)	0.011 (0.013)	−0.001 (0.001)	0.001 (0.013)
Household size	0.001 (0.002)	−0.010 (0.109)	−0.001 (0.004)	− 0.104 (0.097)	0.001 (0,001) *	− 0.156 (0,089) *
Constant		0.001 (0.016)		0.273 (0,114) **		0.137 (0.104)
Number of observations	2796		5397		10,836	

Source: from Demographic and Health Surveys of 1991, 1998, 2004 et 2011

The variables in parentheses are standard deviations *** (**) [*] represent the significance thresholds at 1% (5%) [10%] respectively.

## Discussion

This study has analyzed the effect of immunization aid programs on the probability of children to completely take basic vaccines. We used four DHS carried out in Cameroon in 1991, 1998, 2004 and 2011 by the NIS. To achieve our objectives, we first applied a logistic model to analyze the behavior of full immunization and secondly, Oaxaca’s decomposition to determine the effect of programs from the effect of coefficients or the unexplained portion of the model. Our study has revealed that the vaccination card is the fundamental factor for the full intake of basic vaccines by children. Because children with vaccination card are more than 56 times more likely to be fully immunized (1991). In contrast, other factors come into effect when considering the immunization behavior of children and could call for special attention.

Among the characteristics of the child, with respect to immunization, the child’s birth order is likely to play against him/her. With respect to first children, children of higher birth order are less likely to be fully immunized. Several explanations have been given by various studies to explain this paradox of the child’s birth order which should rather be an asset given the experience gained by the mother. Some suggest that fatigue on the part of mothers due to births and others, an increase in the opportunity cost in terms of time available to mothers which makes them neglect the immunization of the younger children [26]. On the other hand, Blake [27] had already tried to explain this counter-performance through the so-called theory of dilution. According to this theory, as the number of children increases, the resources allocated to each decrease such that children of higher birth order find themselves with less resources than their elders.

Generally, the mother being the person in charge of the child, the effect of her characteristics is also perceptible in our study. They have been captured by factors such as age, religion, level of education, marital status, exposure to media, economic activity and follow-up with prenatal visits. Age has highlighted a certain rejection of society vis-a-vis young mothers (15–19 years). This is due to the fact that they are generally from poor families and are forced to raise their children alone after being rejected by the fathers of the children. In Cameroon, there exist a Ministry of Social Affairs, but which does not have enough resources to support all these cases. Moreover, the Cameroonian legislature has not yet legislated on birth control for mothers deemed indigent. For religion, the focus of reformist religions on miracles is perceived through the negative effect they have on the full immunization of children. As a whole and per period, they reduce the immunization of children up to 43%. Finally, the mother’s level of education is one of the fundamental factors of the immunization of children. This justifies the promotion of the education of women in the world and its consideration as a mechanism to improve the results in relation to child health [30, 31]. Also, this recourse to immunization by older and educated mothers is also the result of advice received during prenatal visits and the good mastery of the child’s immunization schedule which already exists on the prenatal visits card.

In order to ensure the achievement of its objective, which is the total coverage of children with basic vaccines, the EPI has implemented a set of strategies amongst which we have “Service provision strategies”. One of the sections of this strategy is to move from the health facility to find children at least 5 km away and more. This characteristic is found more in rural areas where the density of health facilities is low. As such, in our analysis, residing in rural areas does not have a significant effect on the full immunization of children compared to those who are in urban areas. Furthermore, although the mobile immunization strategy ensures the total intake of basic vaccines by children in rural areas as well as their counterparts of urban areas, it does not however ensure the respect of the immunization schedule of the latter given its importance identified by Bolton, Hussain [34].

All these factors above also facilitate the success of inputs from national and international institutions for the immunization of children. Since 1991, the likelihood of full immunization of children has evolved from 0.368 to 0.561 in 2011. On the other hand, the difference between periods is not significant for the 1998–1991 period. It is however significant for the 2004–1998 and 2011–2004 periods and is higher in the latter. These differences are strongly explained in proportion to the input from institutions accompanying different EPI strategies. As such, it was counted for the 0.071 in unexplained effects the period 2011–2004 which has transited through the characteristics of the mother, 3 provisions that has benefited the EPI partners. These provisions are: the provision of GAVI funds, the implementation of the ACD approach in all HD, catching-up with children within the framework of the AWCMHN and the support of the EPI + UNICEF funds.

## Conclusion

This study evaluated the effect of immunization programs on the probability of children to be fully immunized and has found that the difference in the likelihood of full immunization between periods is mostly explained by the contribution of partners (GAVI, UNICEF, WHO, etc.) supporting immunization as well as strategies to promote immunization. These contributions must be in the direction of mothers given that it is the characteristics of mothers which contribute to this unexplained difference between periods. These characteristics are age, religion, level of education, marital status, the exposure to the media, occupation and the level of follow-up with prenatal check-ups. Our results encourage external contributions in terms of support to national immunization programs which is the EPI in addition to local investments for the sake of program sustainability. However, studies are needed to assess the impact of each program within the scope of the immunization of children less than 5 years of age in order to improve implementation conditions and draw maximum benefits.

## Data Availability

We cannot make the data publicly available, because we were not permitted to do so by the data providers; interested persons should contact the Measure DHS Program (httpd://dhsprogram.com). Data analysis files are however available from the authors upon request.

## Abbreviations

AWCMHN: Action Week on Child and Maternal Health and Nutrition
BCG: Bacillus Calmette-Guèrin
DHS: Demographic and Health Survey
DPT: Diphtheria-Pertusis-Tetanus
DRPHC: Declaration of the Reorientation of Primary Health Care
EPI: Expanded Programme on Immunizations
GAVI: Global Alliance for Vaccines and Immunizations
GIVS: Global Immunization Vision and Strategy
HD: Health District
MAP: Minimum Activity Package
MDGs: Millennium Development Goals
NIS: National Institute of Statistics
OCCEDCA: Organization for the Coordination of the Control of Endemic Diseases in Central Africa
RED: Reach Each District
SDGs: Sustainable Development Goals
SSA: Sub-Saharan Africa
UNFPA: United Nations Fund for Population Activities
UNICEF: United Nations International Children’s Emergency Fund
USAID: United States Agency for International Development
WHO: World Health Organisation

## Footnotes

1 These include MDG4 “Reduce child mortality”, MDG5 “Improve maternal health” and MDG6 “Combat HIV/AIDS, malaria and other diseases”.

2 Tuberculosis, Polio, Dyphteria, Tetanus, Pertussis, Measles and Yellow fever.

3 A single reported case led to a vaccination campaign.

4 In 2011 it is presented as a pentavalent vaccine DTP-HepB-Hib including viral hepatitis B *Haemophilus influenzae* type b.

5 Demographic and Health Surveys generally collect the information on the last 5 years preceding the survey. Since we have four surveys (1991, 1998, 2004 and 2011), taking into account the periods 1998–1991, 2004–1998 and 2011–2004 allows us to assess the effect of all the benefits and implementation of the EPI on the probability of children being fully vaccinated.
